# Patients with borderline hip dysplasia present with inferior patient‐reported outcomes compared to true hip dysplasia

**DOI:** 10.1002/jeo2.70407

**Published:** 2025-09-04

**Authors:** Quentin Karisch, Marco Haertlé, Justus Stamp, Nikolai Ramadanov, Henning Windhagen, Sufian S. Ahmad

**Affiliations:** ^1^ Department of Orthopedic Surgery, Hannover Medical School Diakovere Annastift Hannover Germany; ^2^ Center of Orthopedics and Traumatology, Brandenburg Medical School University Hospital Brandenburg an der Havel Brandenburg an der Havel Germany

**Keywords:** borderline hip dysplasia, DDH, developmental dysplasia of the hip, hip arthroscopy, PAO, patient reported outcome measures, periacetabular osteotomy, PROM

## Abstract

**Purpose:**

The factors influencing patient‐reported outcome measures (PROMs) in individuals with developmental dysplasia of the hip (DDH) remain poorly understood. The aim of this study was to determine the differences in hip‐related PROMs in both borderline and true hip dysplasia.

**Methods:**

A total of 245 patients with symptomatic DDH were enrolled. Hips were divided into either borderline dysplasia (lateral centre‐edge angle [LCEA] 20°–25°) or true dysplasia (LCEA < 20°). PROMs were retrieved from all patients. Linear regression analysis was performed to identify potential factors associated with PROMs. The relationship between PROMs and the characteristic of dysplasia and between patients with DDH and healthy people was assessed.

**Results:**

Patients with borderline hip dysplasia had significantly poorer scores compared to true dysplasia on the University of California and Los Angeles activity scale (UCLA), the Western Ontario and McMaster Universities Osteoarthritis Index (WOMAC), the Merle d'Aubigné and postel score and the German forgotten joint score (G‐FJS). In patients with DDH, all PROMs were significantly lower compared to the control group. In patients with true dysplasia, body mass index (BMI) emerged as the most influential factor affecting the hip disability and osteoarthritis outcome score–physical function shortform (HOOS‐PS), WOMAC, International Hip Outcome Tool 12 (iHOT‐12) and Harris hip score (HHS). In contrast, in patients with borderline dysplasia, radiographic parameters such as anterior wall coverage significantly influenced the WOMAC, iHOT‐12, HHS, modified HHS and G‐FJS, while the LCEA was associated with UCLA and HOOS‐PS scores. Age was identified as a significant predictor in borderline dysplasia.

**Conclusion:**

Patients with borderline dysplasia report poorer PROMs compared to true dysplasia, suggesting a distinct clinical and diagnostic burden in this group of patients. Moreover, the increased mechanical load associated with higher BMI appears to play a greater role only in patients with true dysplasia.

**Level of Evidence:**

Level III.

**Abbreviations**
AC: anterior wall coverage
AI: acetabular index
ALD: anterolateral dysplasia
ANOVA: analysis of variance
a.p.: anterior‐posterior
BMI: body mass index
CI: crossover index
DDH: developmental dysplasia of the hip
EI: extrusion index
G‐FJS: German forgotten joint score
HHS: Harris hip score
HOOS: hip disability and osteoarthritis outcome score
HOOS‐PS: hip disability and osteoarthritis outcome score‐physical function shortform
iHOT‐12: International Hip Outcome Tool 12
LCEA: lateral centre edge angle
mHHS: modified Harris hip score
n.d.: not determined
PC: posterior wall coverage
PLD: posterolateral dysplasia
PMA: postel Merle d'Aubigné Score
PROM: patient‐reported outcome measures
UCLA: University of California and Los Angeles activity scale
WOMAC: Western Ontario and McMaster Universities Osteoarthritis Index

## INTRODUCTION

Developmental dysplasia of the hip (DDH) is a structural hip disorder characterised by insufficient coverage of the femoral head, resulting in joint instability, impaired function and early‐onset osteoarthritis [27].

The literature commonly distinguishes between moderate, borderline and true dysplasia based on lateral centre edge angle (LCEA). A LCEA of 20° to 25° is typically considered indicative of borderline dysplasia, whereas a LCEA of less than 20° defines true dysplasia. Recent evidence indicates that even mild acetabular undercoverage, as seen in borderline DDH, can lead to substantial pain and functional limitations [19].

Furthermore, advances in the understanding of hip dysplasia have enabled a three‐dimensional understanding of phenotypes, which can be broadly categorised as anterolateral, posterolateral or global forms [3, 16, 39].

Despite improvements in neonatal ultrasound screening and a growing comprehension of the pathoanatomical features of DDH, a significant number of cases remain undiagnosed until symptoms of joint instability appear in early adulthood [15].

This delay in diagnosis raises important questions regarding the factors that influence hip joint function in symptomatic adults with DDH. Previous research has shown that overall physical performance—including patient‐reported outcome measures (PROMs), walking speed and stair‐climbing ability—is significantly reduced in individuals with DDH compared to healthy controls [30].

Patients with DDH often report increased mood disturbances, sleep deprivation, difficulties in social relationships and a perceived sense of accelerated aging in relation to healthy peers [18]. Muscle‐induced biomechanical adaptations may further alter joint loading patterns, broadly influencing patients perceptions of joint function [40]. Degenerative radiographic changes, such as cyst formation and osteophyte development, have also been linked to impaired hip joint function in this population [34]. Age appears to be a relevant factor in PROM outcomes, particularly regarding the Western Ontario and McMaster Universities Osteoarthritis Index (WOMAC) and hip disability and osteoarthritis outcome score (HOOS), both of which tend to show deterioration with increasing age [12, 24, 28]. Grammatopoulos et al. compared PROMs between borderline and true dysplasia and found significantly better HOOS scores in the true dysplasia group [13]. However, the literature on this topic remains inconclusive, underscoring the need for further investigation.

The objectives of this study were twofold: primarily, to assess whether PROMs in patients presenting with borderline hip dysplasia are comparable to those in true dysplasia; and secondarily, to identify radiographic and clinical factors that may influence these PROMs. The evaluated outcome measures included the University of California, Los Angeles activity scale (UCLA), HOOS‐physical function short form (HOOS‐PS), WOMAC, International Hip Outcome Tool 12 (iHOT‐12), Harris hip score (HHS), modified Harris hip score (mHHS), postel Merle d'Aubigné score (PMA) and the German forgotten joint score (G‐FJS). We hypothesised that patients with borderline hip dysplasia would report generally poorer PROMs compared to those with true dysplasia.

## METHODS

Between October 2022 and January 2025, a total of 283 patients were considered potentially eligible in the study provided they fulfil the following inclusion criteria: a diagnosis of symptomatic DDH without other concomitant hip pathologies. Of an initial cohort of 283 patients with symptomatic hip dysplasia, 245 met the inclusion criteria and were included in the study. Exclusion criteria included femoroacetabular impingement resulting from asphericity of the femoral head‐neck junction and LCEA ≥ 25°.

In addition, PROMs from 17 individuals without hip complaints were collected and used as a control group for comparison with the dysplasia cohort.

### Radiographic evaluation

In accordance with standard clinical practice, all patients underwent radiological assessment, including a supine anteroposterior (a.p.) pelvic radiograph. Hip morphology was evaluated by measuring the LCEA, acetabular index (AI), extrusion index (EI), AC, posterior wall coverage (PC) and iliac wing rotation. Radiographic measurements were performed according to the methodology described by Tannast et al. [35].

Patients were divided into two groups based on the LCEA: borderline dysplasia (LCEA 20°–25°) and true dysplasia (LCEA < 20°). Additionally, further distinction between anterolateral dysplasia (ALD) and posterolateral dysplasia (PLD) was made [16]. ALD was defined by an LCEA < 25°, a crossover index (CI) < 10%, AC < 25%, and reduced external rotation of the iliac wing. PLD was defined by a CI > 10%, anterior wall coverage (AC) > 25%, a positive posterior wall sign, and increased external rotation of the iliac wing. The distinction into ALD and PLD was performed independently by two experienced hip‐preserving surgeons. In cases of disagreement, a consensus was reached with the involvement of a third hip specialist.

### PROMs and clinical examination

The following PROMs were recorded using a self‐administered questionnaire: UCLA, HOOS‐PS, WOMAC, iHOT‐12, HHS, mHHS, PMA and G‐FJS.

#### UCLA

The UCLA assesses pain, walking ability and joint function [2]. A 10‐point scale is used, where 1 represents the worst possible score and 10 represents the best possible score [2, 23]. Compared to other activity scores, the UCLA scale demonstrates superior reliability and consistent construct validity, without exhibiting floor or ceiling effects [25].

#### HOOS‐PS

The HOOS‐PS is a shortened version of the original HOOS, consisting of only five questions instead of 40, focusing on activity limitations and sports. The score ranges from 0 to 100, with higher values indicating less limitations [9]. Compared to WOMAC, the HOOS has an even better ability to detect changes more reliably [26].

#### WOMAC

The WOMAC is characterised by good reliability, validity and a strong ability to detect changes [7]. The WOMAC consists of 24 questions, divided into three subcategories: WOMAC pain, WOMAC stiffness and WOMAC function/activity. This PROM yields scores ranging from 0 to 100. A score of 100 indicates the worst outcome, while 0 represents the best [7].

#### iHOT‐12

The iHOT‐12 is the short form of the iHOT‐33 [5, 14, 22], designed to assess health‐related quality of life in young, active patients aged 18–60 years with hip problems [22]. It covers four domains: symptoms and functional limitations, sports and activities, work limitations, social, emotional and lifestyle limitations. The iHOT‐12 is measured on a 100‐mm visual analogue scale, resulting in total scores ranging from 0 to 100, with 100 indicating the best possible outcome and 0 the worst [22]. The iHOT‐12 is known for its good reliability, validity and sensitivity to change [22].

#### HHS and mHHS

The HHS consists of 10 questions addressing four domains: pain, function, absence of deformity and range of motion. The patient completes part of the assessment independently, while a medical professional is required for the ‘absence of deformity’ and ‘range of motion’ sections. The HHS ranges from 0 to 100, with the final 9 points requiring assessment by a medical professional. Therefore, when this component is excluded, the maximum achievable score is 91. A score of 0 represents the worst possible outcome, while 100 indicates the best [17]. The HHS is known for its good validity, reliability and sensitivity to change [32], although it exhibits a pronounced ceiling effect [38].

The mHHS is an evolution of the HHS, eliminating the need for a medical professional in the assessment. Due to omitted items, the maximum achievable score is 91, with 0 representing the worst and 91 the best possible outcome [10]. Like the HHS, the mHHS is characterised by high reliability and validity [33].

#### PMA

The PMA assesses three domains: pain, joint mobility and walking ability. It allows for an evaluation of function based on pain and walking ability and can be used to assess postinterventional improvement. The total PMA score ranges from 0 to 18 points, with 0 indicating the worst and 18 the best possible outcome [8]. Although the PMA is widely used in orthopaedic research, no studies have assessed its reliability or validity. Only a modified version of the PMA demonstrated high interrater reliability [36].

#### (G‐)FJS

To address the ceiling effects of the WOMAC, the Forgotten joint score (FJS) was developed. This PROM measures how much patients forget about their treated joint. Scores range from 0 to 100, with 0 indicating the worst and 100 the best possible outcome [6]. The G‐FJS is the validated German translation of the FJS, exhibiting excellent internal consistency, good reliability and no significant floor or ceiling effects [4].

The Beighton score [21] was used to quantify joint flexibility and was assessed using a standardised questionnaire. Patients performed the tests independently under the supervision of a medical professional, who simultaneously administered the questionnaire.

Anthropometric data, including self‐reported height and weight, were collected. Date of birth was recorded to calculate age at the time of assessment.

### Variables and endpoints

The primary objective was to assess whether PROMs in patients presenting with borderline hip dysplasia are comparable to those in true dysplasia. Additionally, PROMs of patients with dysplasia were compared to those of healthy asymptomatic controls with no history of hip pathology.

The secondary objective was to identify factors influencing individual PROMs in patients with symptomatic DDH.

### Statistical analysis

Data were tabulated using Microsoft Excel. Continuous variables are presented as mean ± standard deviation, and categorical variables are presented in binary format. Comparisons between groups were performed using analysis of variance (ANOVA). The post hoc statistical power was calculated using G*Power (version 3.1.9.7). Multivariable linear regression was used to assess the influence of age, sex, side, dysplasia type, body mass index (BMI), Beighton score, LCEA < 20° or LCEA ≥ 20°, LCEA, AI, EI, AC and PC on the individual hip PROMs. Reverse adjustment was subsequently performed. A *p*‐value of <0.05 was considered statistically significant. IBM SPSS Statistics software version 24 was used for the analysis.

### Ethical review committee statement

The study was conducted in accordance with the principles of the Declaration of Helsinki and was approved by the local ethics committee of the Hannover Medical School (10405_BO_K_2022). It was carried out at the Hannover Medical School, Department of Orthopedics, Anna‐von‐Borriesstr. 1‐7, 30625 Hannover.

## RESULTS

A total of 245 patients with DDH were included in the study, while the control group consisted of 17 individuals.

Patients with dysplasia exhibited significantly poorer PROMs compared to the control group, with a post hoc power of 0.96 for both the iHOT‐12 and the mHHS (Table 1).

**Table 1:** Demographic data of dysplasia patients compared to healthy controls.

	All dysplasia patients		Healthy control group		*p*‐Value
	*N*	Mean ± SD	*N*	Mean ± SD	
Number of patients					
		245		17	
Average age (years)					
	245	29.41 ± 7.91	17	28.41 ± 4.52	0.802***
Sex					
	37 males (15%) and 208 females (85%)		11 males (65%) and 6 females (35%)		<0.001*
Body mass index (kg/m^2^)					
	238	25.00 ± 5.17	17	24.10 ± 2.99	0.693***
Side					
	122 right side (50%) and 123 left side (50%)			n.d.	
Radiographic data					
LCEA (°)	245	17.81 ± 7.04		n.d.	
AI (°)	245	14.46 ± 6.57		n.d.	
EI (%)	245	29.06 ± 21.99		n.d.	
AC (%)	245	43.91 ± 27.48		n.d.	
PC (%)	245	86.94 ± 66.76		n.d.	
Joint flexibility					
Beighton	169	2.48 ± 2.65	17	0.65 ± 1.00	0.006***
PROMs					
UCLA	236	5.50 ± 1.98	17	8.35 ± 1.46	<0.001***
HOOS‐PS	230	56.71 ± 22.01	17	99.71 ± 1.21	<0.001***
WOMAC (total)	224	28.42 ± 21.40	17	0.12 ± 0.33	<0.001***
iHOT‐12	234	40.94 ± 21.22	17	96.79 ± 4.47	<0.001***
HHS (without doctors part)	191	59.43 ± 15.28	17	90.76 ± 0.97	<0.001***
mHHS	228	60.25 ± 18.66	17	90.76 ± 0.97	<0.001***
PMA	231	14.31 ± 2.30	17	17.94 ± 0.24	<0.001***
G‐FJS	72	22.05 ± 22.18	17	96.94 ± 4.72	<0.001***

*Note*: *Fisher's exact test. ***Mann–Whitney *U* test.

Abbreviations: AC, anterior wall coverage; AI, acetabular index; EI, extrusion index; G‐FJS, German forgotten joint score; HHS, Harris hip score; HOOS‐PS, hip disability and osteoarthritis outcome score‐physical function shortform; iHOT‐12, International Hip Outcome Tool 12; LCEA, lateral central edge angle; mHHS, modified Harris hip score; n.d., not determined; PC, posterior wall coverage; PMA, Merle d'Aubigné and postel score; PROM, patient reported outcome measures; UCLA, University of California and Los Angeles Activity Scale; WOMAC, Western Ontario and McMaster Universities Osteoarthritis Index.

**Figure 1:**
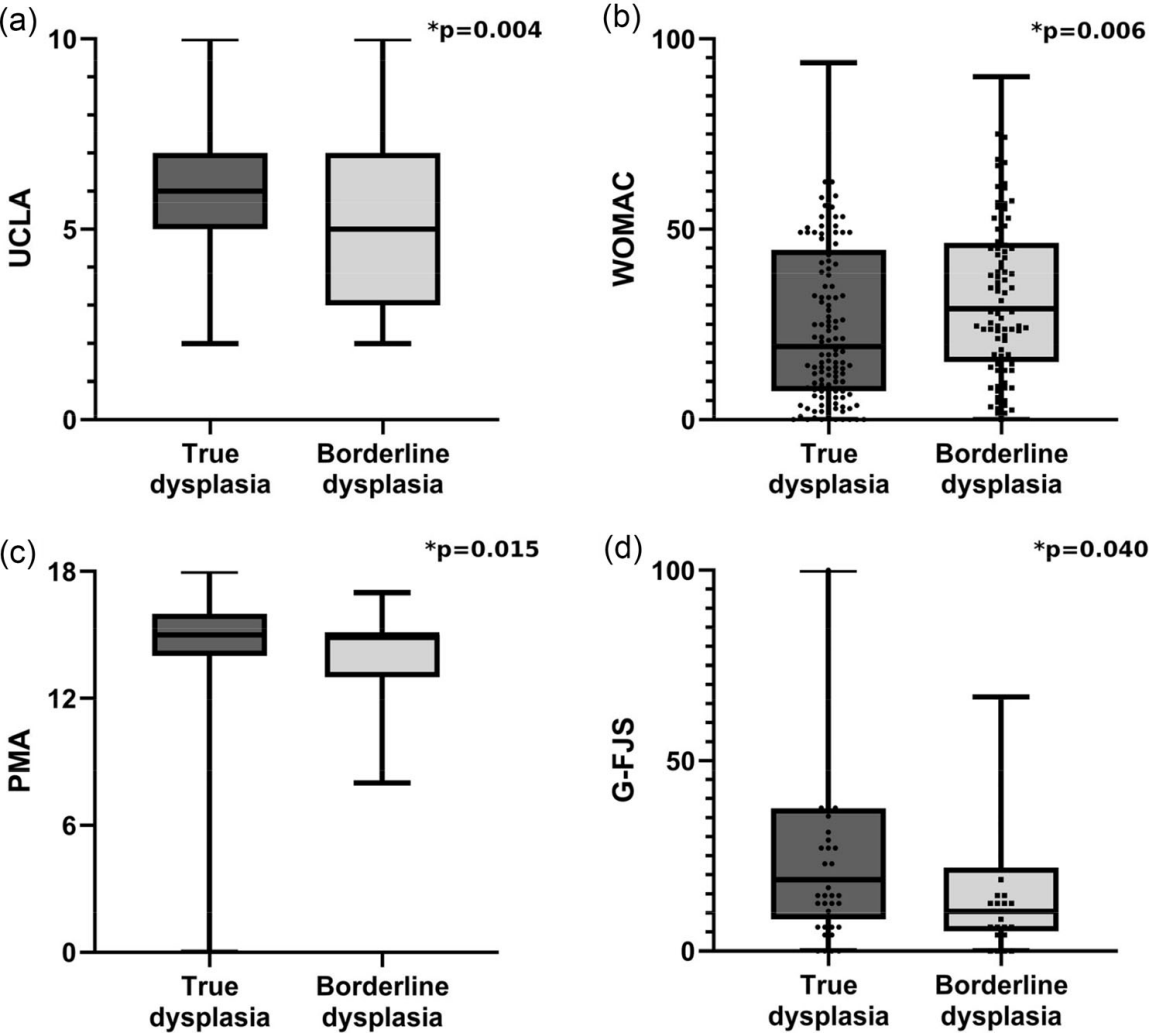
(a) Comparison of the University of California and Los Angeles Activity Scale (UCLA) between true dysplasia (mean = 5.80, standard deviation [SD] = 1.88) and borderline dysplasia (mean = 5.07, SD = 2.06). (b) Comparison of the Western Ontario and McMaster Universities Osteoarthritis Index (WOMAC) between true dysplasia (mean = 25.39, SD = 21.29) and borderline dysplasia (mean = 32.76, SD = 20.91). (c) Comparison of the Merle d'Aubigné and Postel score (PMA) between true dysplasia (mean = 14.59, SD = 2.28) and borderline dysplasia (mean = 13.92, SD = 2.28). (d) Comparison of the German forgotten joint score (G‐FJS) between true dysplasia (mean = 25.87, SD = 24.10) and borderline dysplasia (mean = 16.38, SD = 17.91).

When comparing borderline and true dysplasia, patients with borderline dysplasia exhibited significantly reduced outcomes for UCLA (*p* = 0.004), WOMAC (*p* = 0.006), PMA (*p* = 0.015) and G‐FJS (*p* = 0.040) (Table 2 and Figure 1).

**Table 2:** Descriptive data for true (LCEA > 20°) versus borderline dysplasia (LCEA = 20°–25°).

	True dysplasia		Borderline dysplasia		*p*‐Value
	*N*	Mean ± SD	*N*	Mean ± SD	
Number of patients					
		142/245 (57.96%)		103/245 (42.04%)	
Average age (years)					
	142	29.79 ± 7.19	103	28.88 ± 8.82	0.108***
Sex					
	21 males (15%) and 121 females (85%)		16 males (16%) and 87 females (84%)		1.000*
Body mass index (kg/m^2^)					
	138	25.38 ± 5.48	100	24.48 ± 4.68	0.174***
Side					
	73 right side (51%) and 69 left side (49%)		49 right side (48%) and 54 left side (52%)		0.605*
Joint flexibility					
Beighton	92	2.51 ± 2.68	77	2.44 ± 2.63	0.925***
PROMs					
UCLA	139	5.80 ± 1.88	97	5.07 ± 2.06	0.004***
HOOS‐PS	136	58.61 ± 23.79	94	53.96 ± 18.94	0.068***
WOMAC (total)	132	25.39 ± 21.29	92	32.76 ± 20.91	0.006***
iHOT12	137	43.09 ± 22.16	97	37.90 ± 19.53	0.105***
HHS (without doctors part)	118	60.89 ± 14.58	73	57.08 ± 16.18	0.094**
mHHS	136	61.26 ± 19.71	92	58.74 ± 16.99	0.129***
PMA	136	14.59 ± 2.28	95	13.92 ± 2.28	0.015***
G‐FJS	43	25.87 ± 24.10	29	16.38 ± 17.91	0.040***

*Note*: *Fisher's exact test. **Unpaired *t*‐test; ***Mann–Whitney *U* test.

Abbreviations: G‐FJS, German forgotten joint score; HHS, Harris hip score; HOOS‐PS, hip disability and osteoarthritis outcome score‐physical function shortform; iHOT‐12, International Hip Outcome Tool 12; LCEA, lateral central edge angle; mHHS, modified Harris hip score; PMA, Merle d'Aubigné and postel score; PROM, patient reported outcome measures; UCLA, University of California and Los Angeles activity scale; WOMAC, Western Ontario and McMaster Universities Osteoarthritis Index.

No significant differences in hip PROMs were observed when comparing ALD and PLD.

### Linear regression in true dysplasia

**Figure 2:**
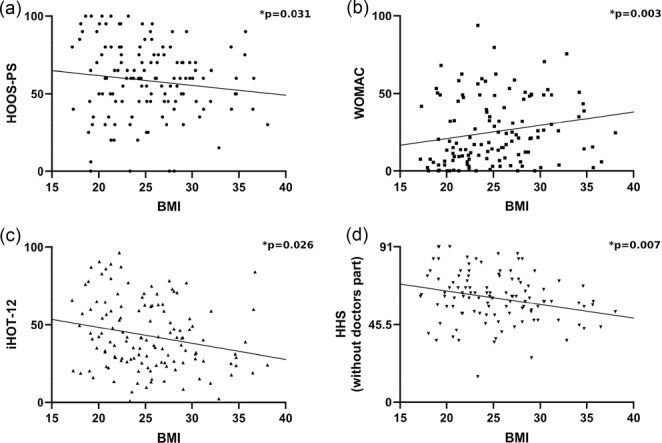
(a) Results of linear regression for the dependent variable Hip disability and osteoarthritis outcome score‐physical function shortform (HOOS‐PS) in the true dysplasia group with body mass index (BMI) as the independent variable (regression coefficient [*B*] = −0.93, *β* = −0.23, lower confidence interval [CI] (*B*) = −1.78, upper CI [*B*] = −0.09). (b) Results of linear regression for the dependent variable Western Ontario and McMaster Universities Osteoarthritis Index (WOMAC) in the true dysplasia group with BMI as the independent variable (*B* = 1.11, *β* = 0.32, lower CI (*B*) = 0.38, upper CI (*B*) = 1.83). (c) Results of linear regression for the dependent variable International Hip Outcome Tool 12 (iHOT12) in the true dysplasia group with BMI as the independent variable (*B* = −0.92, *β* = −0.24, lower CI (*B*) = −1.72, upper CI (*B*) = −0.11). (d) Results of linear regression for the dependent variable Harris hip score without doctors part (HHS) in the true dysplasia group with BMI as the independent variable (*B* = −0.74, *β* = −0.30, lower CI [B] = −1.28, upper CI [*B*] = −0.21).

In patients with true dysplasia, BMI was identified as the primary influencing factor, significantly impacting four out of eight PROMs (Figure 2). The LCEA and AI each influenced three of the eight PROMs, while the radiological parameters EI and PC each influenced one PROM. Specifically, EI positively influenced HOOS‐PS, and PC positively influenced iHOT‐12. Additionally, male sex had a positive influence on UCLA and a negative influence on PMA. ALD was positively associated with PMA (Table 3). The remaining parameters tested, including age, side, AC and Beighton Score, had no significant impact on any PROM (*p* ≥ 0.05).

**Table 3:** Influencing factors in patients with True dysplasia (LCEA < 20°).

Independentfactor	Dependent factor (PROM)	Beta (*β*)	Regression coefficient (*B*)	Confidence interval (*B*)		Sig
BMI	HOOS‐PS	−0.23	−0.93	−1.78	−0.09	0.031
	WOMAC	0.32	1.11	0.38	1.83	0.003
	iHOT12	−0.24	−0.92	−1.72	−0.11	0.026
	HHS	−0.30	−0.74	−1.28	−0.21	0.007
LCEA	WOMAC	0.56	2.37	1.09	3.66	<0.001
	iHOT12	−0.37	−1.72	−3.21	−0.23	0.024
	PMA	−0.39	−0.19	−0.33	−0.39	0.014
AI	WOMAC	0.39	1.45	0.35	2.55	0.010
	mHHS	−0.25	−0.90	−1.64	−0.16	0.018
	PMA	−0.36	−0.15	−0.28	−0.02	0.024
Sex	UCLA	0.22	1.21	0.08	2.34	0.036
0 = f; 1 = m	PMA	−0.25	−1.69	−3.08	−0.31	0.017
EI	HOOS‐PS	0.23	1.07	0.09	2.05	0.032
PC	iHOT‐12	0.23	0.29	0.01	0.58	0.041
Type of dyspl. 0 = ALD; 1 = PLD	PMA	−0.22	−1.44	−2.78	−0.10	0.036

Abbreviations: AI, acetabular index; ALD, anterolateral dysplasia; BMI, body mass index; Dyspl, dysplasia; EI, extrusion index; f, female; HHS, Harris hip score; HOOS‐PS, hip disability and osteoarthritis outcome score‐physical function shortform; iHOT‐12, International Hip Outcome Tool 12; LCEA, lateral central edge angle; m, male; mHHS, modified Harris hip score; PC, posterior wall coverage; PLD, posterolateral dysplasia; PMA, Merle d'Aubigné and postel score; PROM, patient reported outcome measures; Sig, significance; WOMAC, Western Ontario and McMaster Universities Osteoarthritis Index.

### Linear regression in borderline dysplasia

In patients with borderline dysplasia, AC was the most significant radiological parameter, influencing five of the eight PROMs. Age affected four of the PROMs. The degree of LCEA influenced UCLA and HOOS‐PS. Additionally, right‐sided borderline dysplasias showed better iHOT‐12 scores (Table 4). The BMI had no significant influence on PROMs in this group (*p* ≥ 0.05).

**Table 4:** Influencing factors in patients with borderline dysplasia (LCEA 20°–25°).

Independentfactor	Dependent factor (PROM)	Beta (β)	Regression coefficient (B)	Confidence Interval (B)		Sig
AC	WOMAC	0.32	0.43	0.12	0.75	0.008
	iHOT12	−0.23	−0.31	−0.59	−0.03	0.033
	HHS	−0.50	−0.52	−0.76	−0.28	<0.001
	mHHS	−0.48	−0.52	−0.75	−0.29	<0.001
	G‐FJS	−0.59	−0.46	−0.74	−0.19	0.002
Age	UCLA	−0.25	−0.06	−0.11	−0.01	0.033
	HOOS‐PS	−0.23	−0.49	−0.98	−0.003	0.049
	iHOT12	−0.33	−0.80	−1.31	−0.29	0.002
	mHHS	−0.23	−0.48	−0.92	−0.04	0.034
LCEA	UCLA	−0.25	−0.12	−0.23	−0.01	0.031
	HOOS‐PS	−0.32	−1.37	−2.37	−0.37	0.008
Side	iHOT‐12	−0,26	−10.22	−18.66	−1.78	0.002
0 = r; 1 = l						

Abbreviations: AC, anterior wall coverage; G‐FJS, German forgotten joint score; HHS, Harris hip score; HOOS‐PS, hip disability and osteoarthritis outcome score‐physical function shortform; iHOT‐12, International Hip Outcome Tool 12; l, left; LCEA, lateral centre edge angle; mHHS, modified Harris hip score; PROM, patient reported outcome measures; r, right; Sig, significance; UCLA, University of California and Los Angeles Activity Scale; WOMAC, Western Ontario and McMaster Universities Osteoarthritis Index.

Sex, AI, EI, PC and Beighton score had no significant impact on the PROMs (*p* ≥ 0.05).

## DISCUSSION

The most important finding of this study was that patients with borderline hip dysplasia presenting at a hip clinic exhibit poorer PROMs compared to those with true dysplasia. Additionally, BMI was found to have the most significant influence on PROMs in patients with true dysplasia. No differences were observed between the PROMs of patients with ALD and PLD.

Consistent with previous findings [30], this study confirmed significantly poorer PROMs in patients with hip dysplasia compared to a healthy control group.

These results partially confirm the findings of the study by Grammatopoulos et al. [13]. In their study, PROMs were also generally higher in the group with true dysplasia. When comparing the results of this study with theirs, it is evident that although the HOOS‐PS tends to be better in this study for true dysplasia, this difference was not statistically significant. Similar to Grammatopoulos et al., this study found no significant differences regarding HHS, mHHS and iHOT‐12, although these PROMs also tended to be better in true dysplasia, but not significantly. However, this study observed significant differences in four other PROMs: UCLA, WOMAC, PMA and G‐FJS.

An important point when comparing the results of this study with those of Grammatopoulos et al. is the different cutoff value for LCEA. While in this study a cutoff LCEA of 20° was choosen, dividing patients into borderline and true dysplasia, Grammatopoulos et al. used a cutoff LCEA of 15° and only differentiated between milder and more severe forms of dysplasia. Additionally, the group with borderline dysplasia is significantly larger in this study (103 patients) compared to their study (61 patients). Considering the lower cutoff value for LCEA and the influence of LCEA on numerous PROMs demonstrated in this study, this underscores the greater significance of the results of this study [13]. Zhang et al. also investigated potential differences between PROMs in borderline and true dysplasia. However, since their study included only 13 patients with true dysplasia, their result of no differences between the groups is of limited significance [41].

These results suggest that the diagnosis of dysplasia may be difficult in mild cases. Patients with mild dysplasia reported worse PROMs than those with more severe dysplasia. Grammatopoulos et al. conclude in their study that their differences are due to the interaction between dysplasia and impingement in milder forms of dysplasia [13]. Another explanation is that delayed presentation may result from misdiagnosis or misinterpretation of radiographic findings. This highlights the need for increased awareness of hip instability and mild dysplasia, as severe cases are generally easier to diagnose, while milder cases can present a diagnostic challenge due to radiographic appearances that resemble a normal hip. Moreover, this emphasises the impact of hip instability on functional outcomes, suggesting that instability and symptoms may be more important than radiographic findings. The assumption regarding the importance of instability is supported by the fact that borderline patients in the study population seemed to be younger than those with true dysplasia. This age difference has previously been reported in the literature by other authors [29]. Thus, instability not only appears to cause more severe symptoms but may also cause symptoms to manifest at a younger age. Therefore, it is reasonable to involve a specialised hip surgeon in the diagnosis of these cases.

Even with larger sample sizes, this study found no differences between ALD and PLD. This supports the findings of Verhaegen et al. and Fischer et al. [11, 37].

Additionally, BMI was found to significantly affect PROMs, likely due to the increased load on the joint and the corresponding rise in joint reaction forces. Interestingly, the influence of BMI on PROMs was evident only in patients with true dysplasia. Their hips appear to be sensitive to increases in load on the compromised functional surface area. This finding is supported by studies, which also reported significantly poorer preoperative PROMs in patients with higher BMI undergoing hip or knee surgery [20, 31]. In contrast, borderline hips were unaffected by BMI, suggesting that instability is the predominant concern in this subgroup.

Counseling patients on weight loss may be beneficial for reducing joint load; however, the true efficacy of weight loss in treating dysplasia remains to be further investigated, especially in the context of effective weight loss interventions.

Regarding other influencing factors, age also played a key role in borderline dysplasia, as it has been observed in several other studies [12, 24, 28]. Likewise, numerous radiological measurements influenced PROMs in this study. This raises questions regarding the results of the study by Alshaikhsalama et al. Compared to their study, this study had a significantly larger sample size in which the angles were measured by trained professionals rather than by artificial intelligence [1].

This study has several limitations. The most significant one is that the cross‐sectional cohort consists of patients attending a specialised clinic for symptomatic hip dysplasia, where the timing of presentation is not standardised. As a result, the timing of patients' medical consultations and subsequent diagnoses of hip dysplasia influenced the cohort's characteristics. Geographical variations in patient and physician awareness may also affect the timing of presentations at hip clinics. It is reasonable to assume that, with the advancement of hip preservation surgery, these findings reflect the situation in a Western European context. The ability of PROMs to assess subjective hip values is also a limitation. It cannot be ruled out that health perception in dysplasia patients differs from that of healthy individuals, regardless of hip function. Additionally, while most PROMs are commonly used in dysplasia research, they were originally designed for older patients with osteoarthritis [2, 7, 8, 10, 17]. Given that dysplasia patients tend to be younger, this presents a limitation. One exception is the iHOT(−12), which was specifically developed for younger patients [14].

## CONCLUSION

In conclusion, this study demonstrated that borderline hip dysplasia is more debilitating for patients than true hip dysplasia, and that BMI represents a modifiable factor contributing to poorer PROMs in cases of true hip dysplasia. The findings suggest that greater attention is needed to milder forms of dysplasia, as they can be highly limiting for patients. Treatment options such as periacetabular osteotomy (PAO) may be promising in these cases and potentially associated with fewer complications compared to PAO in more severe forms of dysplasia [13]. Additionally, greater emphasis should be placed on BMI and symptoms and functions on patients with true hip dysplasia.

## AUTHOR CONTRIBUTIONS

**Quentin Karisch, Marco Haertlé and Sufian S. Ahmad**: Formulation of the study design, statistical analysis, manuscript writing and revision. **Justus Stamp, Nikolai Ramadanov and Henning Windhagen**: Graphics, manuscript writing and revision.

## CONFLICT OF INTEREST STATEMENT

The authors declare no conflicts of interest.

## ETHICS STATEMENT

Ethics committee of the Hannover Medical School (10405_BO_K_2022). Written informed consent was obtained from all participants prior to inclusion in the study.

## Data Availability

Raw data are available upon request.
